# Supplementary material for: Evaluating user experience with immersive technology in simulation-based education: A modified Delphi study with qualitative analysis
Source: PLoS One. 2023 Aug 2;18(8):e0275766. doi: 10.1371/journal.pone.0275766 (PMC10395907; doi:10.1371/journal.pone.0275766)
Supplement: S2 Text — (RTF) [file pone.0275766.s004.rtf]

Friedman: all pairwise comparisons (Conover)

Critical t (60 df) = 2.000298
Performance metrics (test of skills) vs. Participant Questionnaire of user experience	significant	
(|15| > 13.076094)	P = 0.0253	


Performance metrics (test of skills) vs. Test of knowledge	not significant	
(|-5| > 13.076094)	P = 0.4473	


Performance metrics (test of skills) vs. Quality improvement outcomes	not significant	
(|3| > 13.076094)	P = 0.6479	


Performance metrics (test of skills) vs. Adverse event reports	significant	
(|-23| > 13.076094)	P = 0.0008	


Performance metrics (test of skills) vs. Costs	significant	
(|-35| > 13.076094)	P < 0.0001	


Participant Questionnaire of user experience vs. Test of knowledge	significant	
(|-20| > 13.076094)	P = 0.0033	


Participant Questionnaire of user experience vs. Quality improvement outcomes	not significant	
(|-12| > 13.076094)	P = 0.0714	


Participant Questionnaire of user experience vs. Adverse event reports	significant	
(|-38| > 13.076094)	P < 0.0001	


Participant Questionnaire of user experience vs. Costs	significant	
(|-50| > 13.076094)	P < 0.0001	


Test of knowledge vs. Quality improvement outcomes	not significant	
(|8| > 13.076094)	P = 0.2258	


Test of knowledge vs. Adverse event reports	significant	
(|-18| > 13.076094)	P = 0.0078	


Test of knowledge vs. Costs	significant	
(|-30| > 13.076094)	P < 0.0001	


Quality improvement outcomes vs. Adverse event reports	significant	
(|-26| > 13.076094)	P = 0.0002	


Quality improvement outcomes vs. Costs	significant	
(|-38| > 13.076094)	P < 0.0001	


Adverse event reports vs. Costs	not significant	
(|-12| > 13.076094)	P = 0.0714	


Squared ranks approximate equality of variance test

Chi-square = 6.002337  df = 5   P = 0.306
Pair-wise comparisons:
Critical t (72 df) = 1.993464
Performance metrics (test of skills) and Participant Questionnaire of user experience	variances not different	
(55.730769, 1,431.408254)	P = 0.9384	


Performance metrics (test of skills) and Test of knowledge	variances not different	
(131.692308, 1,431.408254)	P = 0.855	


Performance metrics (test of skills) and Quality improvement outcomes	variances not different	
(515.326923, 1,431.408254)	P = 0.4753	


Performance metrics (test of skills) and Adverse event reports	variances not different	
(610.634615, 1,431.408254)	P = 0.3979	


Performance metrics (test of skills) and Costs	variances not different	
(1,082.807692, 1,431.408254)	P = 0.1359	


Participant Questionnaire of user experience and Test of knowledge	variances not different	
(187.423077, 1,431.408254)	P = 0.7948	


Participant Questionnaire of user experience and Quality improvement outcomes	variances not different	
(459.596154, 1,431.408254)	P = 0.5242	


Participant Questionnaire of user experience and Adverse event reports	variances not different	
(666.365385, 1,431.408254)	P = 0.3565	


Participant Questionnaire of user experience and Costs	variances not different	
(1,138.538462, 1,431.408254)	P = 0.1172	


Test of knowledge and Quality improvement outcomes	variances not different	
(647.019231, 1,431.408254)	P = 0.3706	


Test of knowledge and Adverse event reports	variances not different	
(478.942308, 1,431.408254)	P = 0.5069	


Test of knowledge and Costs	variances not different	
(951.115385, 1,431.408254)	P = 0.1895	


Quality improvement outcomes and Adverse event reports	variances not different	
(1,125.961538, 1,431.408254)	P = 0.1212	


Quality improvement outcomes and Costs	VARIANCES SEEM DIFFERENT	
(1,598.134615, 1,431.408254)	P = 0.0292	


Adverse event reports and Costs	variances not different	
(472.173077, 1,431.408254)	P = 0.5129	


General agreement over all (6) categories with 6 raters per subject

Cohen's kappa (Landis-Koch extension):
Response	Kappa	se	z(for k=0)	Probability	
1	-0.2	0.071611	-2.792848	P = 0.9974	
2	-0.2	0.071611	-2.792848	P = 0.9974	
3	-0.2	0.071611	-2.792848	P = 0.9974	
4	-0.2	0.071611	-2.792848	P = 0.9974	
5	-0.2	0.071611	-2.792848	P = 0.9974	
6	-0.2	0.071611	-2.792848	P = 0.9974	

Combined (Fleiss-Nee-Landis test)
Kappa = -0.2
95% CI: -0.262769 to -0.137231
z (for k = 0) = -6.244998
P > 0.9999
Universal (Berry-Mielke) R
Weighted (linear) kappa = 0.146279
P = 0.0007

General agreement over all (6) categories with 7 raters per subject

Cohen's kappa (Landis-Koch extension):
Response	Kappa	se	z(for k=0)	Probability	
1	0.201754	0.060523	3.33353	P = 0.0004	
2	-0.151899	0.060523	-2.509779	P = 0.994	
3	-0.151899	0.060523	-2.509779	P = 0.994	
4	-0.151899	0.060523	-2.509779	P = 0.994	
5	-0.151899	0.060523	-2.509779	P = 0.994	
6	-0.131841	0.060523	-2.178367	P = 0.9853	

Combined (Fleiss-Nee-Landis test)
Kappa = -0.07565
95% CI: -0.130507 to -0.020793
z (for k = 0) = -2.702876
P = 0.9966
Universal (Berry-Mielke) R
Weighted (linear) kappa = 0.229249
P < 0.0001

General agreement over all (6) categories with 7 raters per subject

Cohen's kappa (Landis-Koch extension):
Response	Kappa	se	z(for k=0)	Probability	
1	-0.166667	0.060523	-2.753785	P = 0.9971	
2	-0.151899	0.060523	-2.509779	P = 0.994	
3	-0.151899	0.060523	-2.509779	P = 0.994	
4	-0.151899	0.060523	-2.509779	P = 0.994	
5	-0.151899	0.060523	-2.509779	P = 0.994	
6	-0.044262	0.060523	-0.731333	P = 0.7677	

Combined (Fleiss-Nee-Landis test)
Kappa = -0.124473
95% CI: -0.180307 to -0.068638
z (for k = 0) = -4.36934
P > 0.9999
Universal (Berry-Mielke) R
Weighted (linear) kappa = 0.186657
P = 0.0001

General agreement over all (7) categories with 7 raters per subject

Cohen's kappa (Landis-Koch extension):
Response	Kappa	se	z(for k=0)	Probability	
0	0.821569	0.060523	13.574542	P < 0.0001	
1	-0.166667	0.060523	-2.753785	P = 0.9971	
2	-0.151899	0.060523	-2.509779	P = 0.994	
3	-0.151899	0.060523	-2.509779	P = 0.994	
4	-0.151899	0.060523	-2.509779	P = 0.994	
5	-0.151899	0.060523	-2.509779	P = 0.994	
6	-0.131841	0.060523	-2.178367	P = 0.9853	

Combined (Fleiss-Nee-Landis test)
Kappa = -0.077701
95% CI: -0.128712 to -0.02669
z (for k = 0) = -2.98544
P = 0.9986
Universal (Berry-Mielke) R
Weighted (linear) kappa = 0.269076
P < 0.0001
