# Supplementary material for: Evaluating user experience with immersive technology in simulation-based education: A modified Delphi study with qualitative analysis
Source: PLoS One. 2023 Aug 2;18(8):e0275766. doi: 10.1371/journal.pone.0275766 (PMC10395907; doi:10.1371/journal.pone.0275766)
Supplement: S3 Text — (DOCX) [file pone.0275766.s005.docx]

Field notes

1^st^ question - Top 4 that appeared

J

- repetition is the most important but it is important for general learning not just simulation
- Fidelity high up – with enjoyment and immersion, then debrief
- Psychological safety – letting them know it’s a safe space if they make a mistake – helps with engagement and immersion – they go together

DM

- agrees with top 4

JK

- agrees – would choose those 4
- think whilst Learning objectives are important – you usually learn things not defined in learning objectives

V

- agrees
- makes sense – but expected team work higher

DM

- engagement is instantaneous feedback about simulation
- then can consolidate learning with debrief
- rest comes after

2^nd^ question - what factors do you think are important

SK

- depends what you do you define as important
- simulation as a concept can be a challenge
- cost effectiveness is hard to measure but is very important to collect data on

V

- patient safety is very important
- and proving worth Is important – so safety is a way to show this

SK

- easy to measure confidence but feels like a not good outcome as doesn’t reflect real world
- so patient safety is key to measure but hard as a lot of other factors and bad outcomes don’t occur often – so both are important
- Patient safety more so – but very hard to measure

SH

- Both interlinked – confidence with clinical skills should lead to patient safety

V

- Doesn’t agree with top 4 as user stress levels should be higher so no

J

- This tech is novel and is useful compared to standard methods – so immersion and testing how useful the technology is is important
- Therefore doesn’t 100% agree as should be higher

When cost effectiveness was added the agreement rate increased

3^rd^ question – how could we measure it

Most acceptable is to ask what the person thought

SK

- yes and no – good as it is easy to ask what they think – but SK thinks whether the tech has made a difference after the session should be measured too
- best measure is quality improvement and adverse events

barriers

SH

- the first 4 factors are the most important so would agree with this
- engagement they especially agree with
- eg – CBT is good but if used poorly done - if engagement and confidence is not there then it doesn’t work – can be damaging
- time – cost effectiveness of time and getting it done right is important to consider
- cost can include cost of time not just money
