# Supplementary material for: Evaluating user experience with immersive technology in simulation-based education: A modified Delphi study with qualitative analysis
Source: PLoS One. 2023 Aug 2;18(8):e0275766. doi: 10.1371/journal.pone.0275766 (PMC10395907; doi:10.1371/journal.pone.0275766)
Supplement: Attachment — Submitted filename: Review Comments.docx [file pone.0275766.s006.docx]

Evaluating user experience with immersive technology in simulation-based education: a modified Delphi study with qualitative analysis

The subject matter of this essay is novel. The structure of the article is correct, but there are still many problems. The correct structure does not appear in the correct content which makes the reader confused when reading. The research process is too rough and the results appear too abruptly. In my opinion this article is more like a research outline at this stage. The paper is not ready for publication until the following issues are resolved.

1. The Background should be populated with detailed examples such as pictures of realistic immersive technologies being implemented in medical education. In this way, the richness of the Background can be improved.
2. In the Materials and Methods section, "A modified Delphi method based on the traditional method" should be briefly compared again with the differences between the two. This is to highlight the advantages of the improved Delphi method. This will enable the reader to better understand the improved Delphi method.
3. The research method in Materials and Methods is too homogeneous. The single method may not be able to support the content of the study. It is recommended to expand the research methods to enrich the research content and improve the reliability of the research.
4. The description of Materials in Materials and Methods is missing or too brief. Also the instruments used in the study. The location of the interview process. How the questionnaire was generated in the survey.
5. Participants should give details about the overall population, sample size and participants.
6. The data collection and analysis section is too simple or crude. The Data collection and analysis section is too crude and makes it difficult for the reader to understand the process of the study.
7. The data collection process in Delphi Round 1 is too simple and should be described in detail here. The questionnaire used in the study should be presented in more detail to confirm the reliability of the questionnaire.
8. Delphi Round 1 should include a brief citation and introduction of representative literature from the 246 papers analyzed.
9. The values obtained with StatsDirect version 3.3.5 as mentioned in Delphi Round 2 should be shown here.
10. Both the zoom online meeting and the zoom polling tool in Delphi Round 3 should be described in detail. They are eventually shown here through images and other forms.
11. The results in this article are too abrupt. The study lacks a process that should detail how the resultant data were obtained. The description of the data for the results is also not adequate. The outcome data gives the reader the impression that it appeared out of thin air. This makes it difficult for the reader to understand in detail how the results of the study were derived.
12. The conclusions should be supplemented with appropriate study data to better substantiate the findings.
